# Comparison of Clinical Efficacy and Postoperative Outcomes of Ultrasound-Guided Lateral Sagittal Infraclavicular and Costoclavicular Brachial Plexus Blocks: A Retrospective Cohort Study

**DOI:** 10.3390/diagnostics16203274

**Published:** 2026-10-09

**Authors:** Ahmet Demirdağ, Sezen Özkaya, Ahmet Aksu

**Affiliations:** 1 Department of Anesthesiology and Reanimation, Faculty of Medicine, Fırat University, 23119 Elazığ, Türkiye; aaksu@firat.edu.tr; 2 Department of Anesthesiology and Reanimation, Bingöl State Hospital, 12000 Bingöl, Türkiye; sezenaylineren@hotmail.com

**Keywords:** costoclavicular block, lateral sagittal infraclavicular block, ultrasound-guided regional anesthesia, brachial plexus, procedural efficiency, postoperative analgesia

## Abstract

**Background/Objectives:** The costoclavicular (CCB) and lateral sagittal infraclavicular (ICB) approaches provide ultrasound-guided access to the cords of the brachial plexus. Comparative real-world evidence on imaging efficiency, block dynamics, and postoperative analgesia remains limited. We compared procedural and analgesic outcomes between these approaches. **Methods:** This retrospective two-center cohort included patients undergoing elective upper-extremity surgery under ultrasound-guided CCB or ICB between April and October 2025. Specialists at both centers had comparable experience with both techniques (approximately 4 years). The primary outcome was total procedure time; secondary outcomes included component times, exact block-onset times recorded during close continuous specialist observation, postoperative pain, rescue analgesia, satisfaction, and discharge time. All patients received the same local-anesthetic dose and volume. **Results:** Sixty patients were analyzed (29 CCB; 31 ICB). Total procedure time was shorter with ICB (median, 75 vs. 138 s; CCB-ICB Hodges–Lehmann median difference, 65 s; 95% CI, 23–110 s; *p* < 0.001), as were imaging and application times. Motor- and total-block onset were faster with ICB, whereas sensory-block onset did not differ significantly. Pain scores at 6, 12, and 24 h, rescue analgesia, analgesia duration, satisfaction, and discharge time did not differ significantly. Hemidiaphragmatic paralysis is generally asymptomatic and was not systematically assessed; no local anesthetic systemic toxicity occurred. **Conclusions:** ICB was associated with statistically shorter procedural and selected block-onset times than CCB. However, the absolute difference in total procedure time was modest, and its clinical importance remains uncertain. The absence of corresponding differences in sensory-block characteristics and patient-centered postoperative outcomes means that these findings should not be interpreted as demonstrating the overall clinical superiority of ICB. Prospective randomized studies incorporating predefined clinically important differences and adequately powered patient-centered outcomes are required.

## 1. Introduction

Ultrasound guidance permits real-time identification of neural and vascular structures during brachial plexus blockade for upper-extremity surgery [1]. Infraclavicular approaches can provide surgical anesthesia and postoperative analgesia [2,3].

The lateral sagittal infraclavicular block (ICB) deposits local anesthetic around the cords of the brachial plexus near the axillary artery [3]. The costoclavicular block (CCB) targets the same plexus more medially, where the cords are clustered within the costoclavicular space and can often be visualized in a single sonographic window [4,5,6]. Comparative studies have reported mixed findings for imaging time, needle execution, onset, and block quality [7,8,9,10,11,12,13].

Randomized comparisons of CCB and ICB have produced divergent findings regarding procedural performance and block onset [8,9,10,11,12,13]. A recent randomized, assessor-blinded study in *Diagnostics* found similar clinical efficacy and no consistent difference in near-infrared spectroscopy-derived tissue oxygenation between the approaches [14]. However, real-world two-center evidence on sonographic acquisition, total procedure time, block dynamics, and postoperative recovery remains limited. We hypothesized that procedural efficiency and block dynamics would differ between approaches in routine practice. We therefore compared total block procedure time as the primary outcome and evaluated block dynamics, postoperative pain, rescue analgesia, satisfaction, and recovery in a retrospective two-center cohort.

## 2. Materials and Methods

### 2.1. Study Design and Reporting

This retrospective two-center cohort study was conducted in accordance with the Declaration of Helsinki and is reported according to the Strengthening the Reporting of Observational Studies in Epidemiology (STROBE) statement [15]. The protocol was approved by the Non-Interventional Research Ethics Committee of the Faculty of Medicine, Fırat University (decision no. 2025/16-18; 6 November 2025). The committee waived additional study-specific written informed consent because the analysis used existing clinical records. Written consent for anesthesia and surgery had been obtained as part of routine care. A completed STROBE checklist is submitted as Supplementary File S1.

### 2.2. Setting and Participants

Records were retrieved from Fırat University Hospital in Elazığ and Bingöl State Hospital in Bingöl for the period between April and October 2025. Eligible patients underwent elective upper-extremity surgery under ultrasound-guided CCB or lateral sagittal ICB as the primary anesthetic and had a complete standardized block-assessment record; all included patients were of ASA physical status I–III. No upper or lower age limit was applied during record selection, and the analyzed cohort therefore spanned 12–83 years. Patients were excluded if they received general anesthesia or a combined anesthetic technique, underwent surgery at more than one operative site in the same session, or had an incomplete standardized block-assessment record.

**Figure 1:**
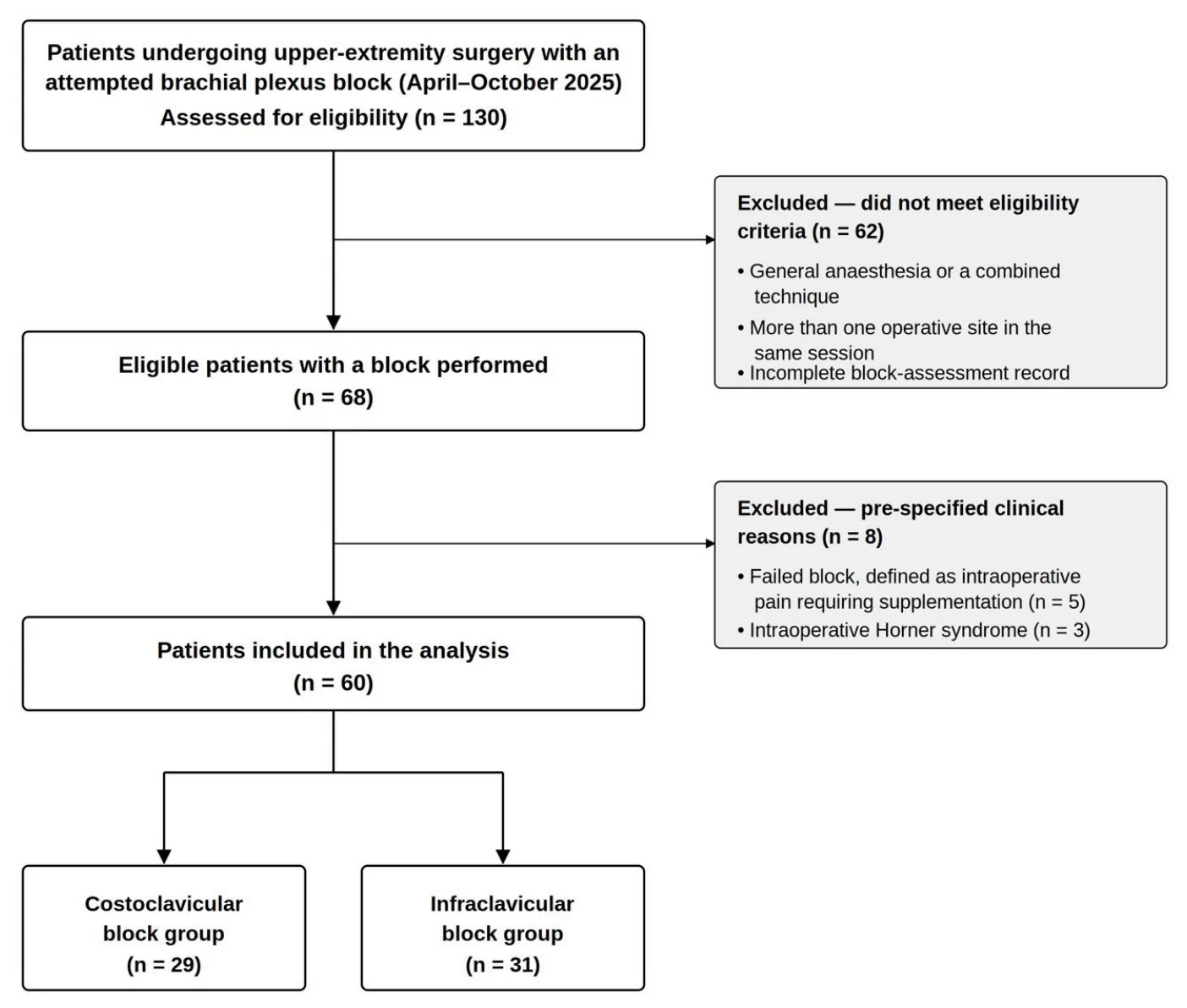
Participant flow through the study (STROBE diagram). Of 130 patients assessed for eligibility, 62 were excluded because of general anesthesia or a combined technique, more than one operative site in the same session, or an incomplete block-assessment record. Eight were excluded after block performance (failed block: CCB, *n* = 2; ICB, *n* = 3; Horner syndrome: CCB, *n* = 2; ICB, *n* = 1), leaving 60 patients for analysis (29 CCB and 31 ICB).

During screening, 130 patients underwent upper-extremity surgery in which a brachial plexus block was attempted. Sixty-two patients did not meet the eligibility criteria, because they received general anesthesia or a combined technique, had more than one operative site in the same session, or had an incomplete block-assessment record. Of the remaining 68 patients in whom a block was performed, eight were excluded for prespecified clinical reasons: five failed blocks, defined as intraoperative pain requiring supplementation, and three cases of intraoperative Horner syndrome. These events are reported at the screening level rather than being interpreted as absent from the analytic cohort (Figure 1).

### 2.3. Block Techniques and Local Anesthetic

All blocks were performed under ultrasound guidance with a high-frequency linear transducer, using an in-plane needle approach. The anesthesiology specialists who performed CCB and ICB at the two participating centers had comparable experience with both techniques (approximately 4 years). For the ICB, the transducer was placed in the parasagittal plane in the infraclavicular fossa and local anesthetic was deposited around the axillary artery at the level of the cords. For the CCB, the transducer was positioned just below the clavicle to visualize the three cords lateral to the axillary artery within the costoclavicular space, and local anesthetic was injected to surround the cluster. Every patient received a fixed volume of 20 mL, comprising 15 mL of 0.5% bupivacaine and 5 mL of 2% prilocaine.

The same local-anesthetic dose and volume were intentionally used for all patients to standardize exposure and minimize dose-volume confounding between techniques. Accordingly, the study was not designed to compare different dose-volume regimens. The selected 20 mL institutional regimen was intended to balance onset and analgesic duration while limiting total dose; prior dose-finding work provides context for volume-dependent block performance [16].

### 2.4. Outcomes and Measurements

The primary outcome was total block procedure time, defined as imaging time plus block application time. Imaging time was the interval from probe placement to identification of the target sonoanatomy; block application time was the interval from skin puncture to completion of local-anesthetic injection. Secondary outcomes were needle passes; onset times of sensory, motor, and total block; motor-block duration; duration of analgesia; postoperative pain; rescue-analgesic requirement; patient and surgeon satisfaction; and time to discharge.

Block characteristics were extracted from structured anesthesia records completed prospectively as part of routine practice. Sensory block was assessed by loss of cold sensation over the relevant dermatomes, and motor block by the institutional modified Bromage-type ordinal scale for the hand and forearm; complete motor block corresponded to the highest category on that scale. From completion of local-anesthetic injection, the responsible anesthesiology specialist closely and continuously observed the patient and repeated sensory and motor examinations until complete block criteria were achieved. Rather than using fixed 5 min intervals, the exact elapsed time at which each criterion was first met was recorded. Sensory- and motor-block onset were the exact intervals from completion of injection to the respective complete-block criteria; total-block onset was the exact interval until both criteria had been met. Block success was defined as completion of surgery under the planned block without intraoperative anesthetic supplementation. Needle-pass counts were taken from the structured procedural record. Postoperative pain was recorded using a 0–10 VAS at 6, 12, and 24 h, and patient and surgeon satisfaction were recorded on 0–10 scales. Assessments were made by the responsible clinical specialist rather than an independent blinded observer.

Safety outcomes were obtained from the intraoperative and postoperative anesthesia records. The records were reviewed for clinically apparent hemidiaphragmatic paralysis and local anesthetic systemic toxicity throughout the recorded perioperative observation period.

Postoperative analgesia was administered as rescue treatment according to a standardized stepwise protocol: intravenous paracetamol 1 g as first-line therapy, intravenous dexketoprofen 50 mg as second-line therapy, and tramadol 50–100 mg for pain not relieved by non-opioid agents or a VAS score ≥ 4; meperidine could be used when tramadol was contraindicated according to institutional practice. All postoperative analgesic drugs, doses, and administration times were recorded. Because opioid use was infrequent and cumulative opioid-equivalent exposure was not prespecified, rescue-analgesic requirement was analyzed as the proportion of patients receiving at least one rescue agent within each postoperative interval.

### 2.5. Statistical Analysis

Analyses were performed using IBM SPSS Statistics, version 21 (IBM Corp., Armonk, NY, USA). Continuous variables were assessed for normality using the Shapiro–Wilk test and visual inspection. Normally distributed variables are presented as mean ± standard deviation and were compared using independent-samples *t*-tests. Non-normally distributed variables are presented as median (interquartile range) and were compared using the Mann–Whitney U test. Categorical variables are presented as *n* (%) and were compared using the chi-square test or Fisher’s exact test, as appropriate. All tests were two-sided, and *p* < 0.05 was considered statistically significant.

For the primary outcome, the between-group effect was quantified using the Hodges–Lehmann median difference with its 95% CI, in addition to the Mann–Whitney U test. No a priori sample-size calculation was performed because the available sample was fixed by the retrospective study period and completeness criteria. Secondary analyses were exploratory, no formal correction for multiple comparisons was applied, and non-significant results were not interpreted as evidence of equivalence or non-inferiority.

## 3. Results

### 3.1. Participants and Baseline Characteristics

Of 130 screened patients, 60 met the eligibility criteria and had complete block-assessment records: 29 received a CCB and 31 received an ICB. Screening and allocation are summarized in Figure 1. At the screening level, completed blocks were recorded in 29 CCB and 31 ICB patients; failed blocks occurred in 2 CCB and 3 ICB patients, and Horner syndrome in 2 CCB and 1 ICB patients. These events are reported at the screening level because they were excluded from the analytic cohort.

No statistically significant between-group differences were detected in the measured demographic or clinical characteristics or in the distribution of surgical sites (Table 1). At both centers, the anesthesiology specialists performing CCB and ICB had comparable experience with both techniques (approximately 4 years).

### 3.2. Block Performance and Dynamics

**Figure 2:**
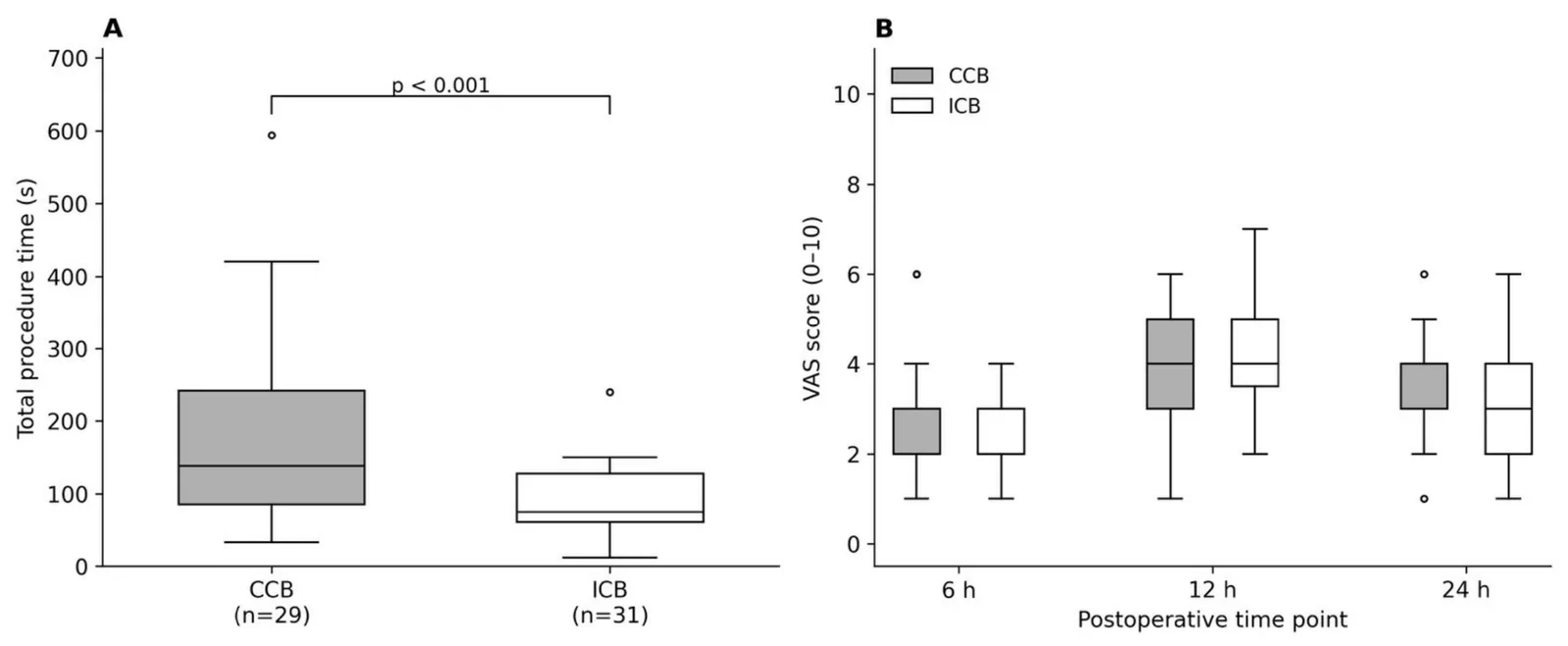
Between-group comparison of (**A**) total block procedure time and (**B**) postoperative Visual Analog Scale (VAS) pain scores at 6, 12, and 24 h. Boxes show the median and interquartile range; whiskers show 1.5 times the interquartile range; circles show observations beyond those limits. CCB, costoclavicular block; ICB, infraclavicular block.

In unadjusted comparisons, imaging time, block application time, and total procedure time were shorter in the ICB group (Table 2). Median total procedure time, the primary outcome, was 75 s with ICB and 138 s with CCB (CCB-ICB Hodges–Lehmann median difference, 65 s; 95% CI, 23–110 s; *p* < 0.001; Figure 2A). Needle-pass counts did not differ significantly. Motor-block and total-block onset were faster with ICB, whereas no statistically significant difference was detected in sensory-block onset. No statistically significant between-group differences were detected in motor-block duration, duration of analgesia, patient or surgeon satisfaction, or time to discharge.

### 3.3. Perioperative Variables, Postoperative Pain, and Rescue Analgesia

No statistically significant between-group differences were detected in heart rate or peripheral oxygen saturation across the recorded perioperative time points (Supplementary Table S1). Mean arterial pressure was lower in the CCB group at the intraoperative and postoperative measurements, but no hypotension requiring vasopressor treatment was documented. Operative duration did not differ significantly.

Postoperative VAS scores were low to moderate and did not differ significantly at 6, 12 or 24 h (Table 3; Figure 2B). The proportion of patients requiring at least one rescue analgesic was also similar across postoperative intervals (Table 4).

### 3.4. Complications and Safety Observations

Among the 68 patients in whom a block was performed during screening, five failed blocks and three cases of Horner syndrome were recorded before exclusion. No clinically apparent hemidiaphragmatic paralysis or local anesthetic systemic toxicity occurred in any of these 68 patients during the recorded perioperative observation period. Because failed blocks and Horner syndrome were excluded before the primary 60-patient analysis, those events are reported at the screening level and comparative safety should not be inferred from the analytic cohort alone.

## 4. Discussion

In this retrospective two-center cohort, ICB was associated with shorter imaging, application, and total procedure times and faster motor- and total-block onset than CCB. No statistically significant between-group differences were detected in sensory onset, duration of analgesia, postoperative pain, rescue analgesia, satisfaction, or discharge time. No local anesthetic systemic toxicity was documented during the recorded perioperative observation period; however, diaphragmatic function was not specifically or systematically assessed. These findings must be interpreted in the context of the observational design and modest sample size.

The literature comparing CCB and ICB remains mixed. The costoclavicular space offers an appealing sonographic target because the cords are clustered and relatively superficial [4,5,6], and some trials have reported favorable onset or procedural performance with CCB [8,10,11,12,13]. Other randomized evidence and meta-analytic findings suggest broadly similar performance between approaches [7,9]. A recent randomized, assessor-blinded study found comparable clinical block efficacy and no consistent difference in tissue-oxygenation profiles [14]. The present study addresses a different question by examining imaging and application efficiency in routine clinical practice; its unadjusted finding of faster ICB performance should not be read as definitive evidence of technical superiority.

Because the specialists performing both techniques had comparable experience at both centers (approximately 4 years), a systematic imbalance in reported experience is unlikely to explain the observed procedural-time difference. The difference may instead reflect approach-specific features of sonographic target acquisition and needle advancement. Nevertheless, the retrospective design and absence of operator identifiers prevent definitive attribution of the observed effect to the block technique alone.

The costoclavicular space is part of the proximal pathway described as the ‘axillary tunnel,’ a near-rigid-surrounding anatomical arrangement that influences longitudinal local-anesthetic spread around the neurovascular bundle [17]. Although the cords are clustered at this level [4,6], the neurovascular bundle does not constitute a single unrestricted space: imaging and anatomical studies have identified compartmentalization at both costoclavicular and lateral infraclavicular levels [18]. A septum between the lateral cord and the medial and posterior cords may impede complete spread at this point after a single injection [19]. If the operator seeks visible spread between the neural elements, needle repositioning may then take additional time and require further redirections. This remains a hypothesis for our procedural findings because injectate distribution and the reasons for individual needle redirections were not recorded. The same compartmentalization cannot by itself establish why CCB took longer than ICB, as compartments occur at both sites [18].

Needle trajectory may offer another possible explanation. A cadaver study identified cutaneous and pectoral nerves close to the costoclavicular needle path and reported proximity to the lateral cord [20]. Careful needle-tip visualization and adjustment of the trajectory could therefore add time when the acoustic window or neural and vascular relationships are less favorable. We did not record the specific acoustic or anatomical obstacles encountered in individual patients, so this proposed mechanism cannot be confirmed in our cohort. Moreover, controlled studies have reported shorter or comparable performance time with CCB [11,12,21]. Dost et al. compared a single-injection costoclavicular technique with a triple-injection lateral sagittal technique, making injection strategy a plausible contributor to their procedural comparison [12]. Differences in injection strategy, timing definitions, operator technique, patient anatomy, and satisfactory-spread endpoints may help explain variation across studies. The pediatric population in Yayik et al. [21] also limits direct extrapolation to this cohort, whose age range was 12–83 years.

The estimated median between-group difference in total procedure time was 65 s (95% CI, 23–110 s) in favor of ICB. A difference of approximately one minute is unlikely to alter analgesic quality or discharge timing for an individual patient, although it could have modest operational relevance when repeated across a high-volume regional-anesthesia service. Given the non-randomized observational design, this estimate is best viewed as an unadjusted procedural-performance signal rather than definitive evidence of an intrinsic advantage of ICB.

Motor and total block onset appeared faster with ICB, whereas sensory onset was similar. Exact onset times were recorded during close continuous observation by the responsible specialist, providing greater temporal resolution than fixed-interval assessment. Nevertheless, sensory and motor evaluation was clinical and unblinded, so between-observer judgment and residual measurement error may have influenced the recorded onset times. The onset findings should therefore be interpreted as clinical temporal associations rather than mechanistic evidence.

The similar VAS trajectories, analgesia duration, and rescue-analgesic requirements suggest that, once an effective block is established at the infraclavicular level, analgesic performance may be comparable between approaches. Every patient received the same local-anesthetic dose and 20 mL volume, reducing dose-volume confounding in the between-group comparison; however, the study did not test whether different dose-volume regimens modify performance [16]. The study was not powered for equivalence or non-inferiority, so the absence of statistically significant differences does not demonstrate equivalence. A prospective trial using a prespecified minimal clinically important difference is needed.

Across all 68 patients in whom a block was performed, no local anesthetic systemic toxicity was documented during the recorded perioperative observation period. However, diaphragmatic function was not specifically evaluated using a standardized method, such as ultrasonographic assessment of diaphragmatic excursion or thickening fraction. Therefore, the occurrence of subclinical or clinically unrecognized hemidiaphragmatic paralysis could not be reliably determined.

Failed blocks and patients who developed Horner syndrome were excluded before the primary comparative analysis and were therefore retained only in the screening-level data. Horner syndrome may indicate unintended proximal spread of local anesthetic and could theoretically be associated with spread toward structures involving the phrenic nerve, although its presence alone does not establish phrenic nerve involvement. Exclusion of these patients may therefore have selected an analytic cohort with fewer manifestations of proximal local-anesthetic spread, limiting interpretation of comparative safety outcomes. Future prospective studies should include standardized diaphragmatic assessment and prespecified complication surveillance [22,23].

### Limitations

Several limitations qualify these findings. Treatment allocation reflected clinical practice rather than randomization, leaving the analysis vulnerable to measured and unmeasured confounding. Although both approaches were performed by specialists with comparable experience at both centers (approximately 4 years), operator identifiers were unavailable; operator-level clustering and center effects therefore could not be modeled.

The complete-case analytic cohort excluded failed blocks and patients who developed Horner syndrome, introducing selection bias and limiting the comparative assessment of both effectiveness and safety. Because Horner syndrome may represent unintended proximal local-anesthetic spread, excluding these patients before the primary analysis may have resulted in underrepresentation of proximal-spread events in the analyzed cohort. Furthermore, diaphragmatic function was not specifically or systematically evaluated, and no standardized ultrasonographic assessment was performed. Consequently, the incidence of subclinical or clinically unrecognized hemidiaphragmatic paralysis could not be determined from the available data.

Although no local anesthetic systemic toxicity was documented during the recorded observation period, the modest sample size precludes reliable estimation of the risk of this and other uncommon complications. The study was not powered for equivalence or non-inferiority of analgesic outcomes. The age range was wide (12–83 years), and age-stratified analysis was not feasible. Although exact onset times were recorded under close observation, assessments were performed by unblinded specialists and included clinical judgment, permitting observer bias. The same local-anesthetic dose and 20 mL volume were intentionally used for all patients; therefore, findings do not address other dose-volume regimens. Finally, injectate-spread patterns, anatomical obstacles, and the reason for each needle redirection were not recorded, preventing direct testing of the proposed anatomical explanations.

## 5. Conclusions

In this retrospective two-center cohort of patients undergoing upper-extremity surgery, ultrasound-guided ICB was associated with shorter imaging, application, and total procedure times and faster motor- and total-block onset than CCB. However, the absolute between-group difference in total procedure time was approximately one minute, and its clinical importance remains uncertain. No statistically significant differences were detected in sensory onset, duration of analgesia, postoperative pain, rescue analgesia, satisfaction, or discharge time. Accordingly, the observed procedural differences should not be interpreted as evidence of the overall clinical superiority of ICB. No local anesthetic systemic toxicity was documented during the recorded perioperative observation period. Because diaphragmatic function was not systematically assessed, this study cannot establish the incidence of hemidiaphragmatic paralysis or compare the safety of the two approaches. The non-randomized design and modest sample size limit the interpretation of these findings, particularly for analgesic outcomes. Prospective randomized studies incorporating predefined clinically important differences, blinded assessment, and adequately powered patient-centered outcomes are required.

## Figures and Tables

**Table 1:** Baseline demographic, clinical and procedural characteristics of the two groups.

Variable	Costoclavicular (*n* = 29)	Infraclavicular (n = 31)	*p*
Age, years—median (IQR) [range]	37 (25–50) [17–83]	45 (17–62) [12–83]	0.935
Weight, kg	73.6 ± 10.4	72.6 ± 16.1	0.780
Height, m	1.68 ± 0.09	1.70 ± 0.10	0.385
Body mass index, kg/m^2^	26.2 ± 4.2	25.0 ± 5.4	0.366
Sex, male/female—*n* (%)	19 (65.5)/10 (34.5)	21 (67.7)/10 (32.3)	1.000
ASA class, I/II/III—*n*	7/15/7	7/17/7	0.971
Current smokers, *n* (%)	14 (48.3)	15 (48.4)	1.000
Any comorbidity, *n* (%)	10 (34.5)	11 (35.5)	1.000
Surgical site, *n*			0.169
Hand/wrist	15	21	
Forearm (radius/ulna)	8	5	
Elbow	1	4	
Humerus	3	1	
Implant removal	2	0	

Continuous variables are shown as median (interquartile range [IQR]) or mean ± standard deviation according to distribution; the age range (minimum–maximum) is given in square brackets. Categorical variables are counts or *n* (%). Comparisons used the Mann–Whitney U test, independent-samples *t* test, chi-square test or Fisher exact test as appropriate. No age limit was applied at record selection; the cohort therefore included adolescent and older patients (see Section Limitations). ASA, American Society of Anesthesiologists physical status.

**Table 2:** Block performance metrics and clinical block characteristics.

Variable	Costoclavicular (*n* = 29)	Infraclavicular (n = 31)	*p*
Imaging time, s	15 (10–45)	10 (3–15)	0.002
Block application time, s	105 (70–205)	60 (53–120)	0.005
Total procedure time, s	138 (85–242)	75 (61–128)	<0.001
Hodges–Lehmann median difference (CCB − ICB), 95% CI	65 s (23 to 110)		
Needle passes, n	1 (1–2)	1 (1–1)	0.380
Sensory block onset, min	15 (10–15)	15 (10–15)	0.860
Motor block onset, min	16 (15–20)	15 (13–15)	<0.001
Total block onset, min	19 (15–20)	15 (13–17)	0.002
Motor block duration, h	11.0 ± 3.5	11.0 ± 2.2	0.981
Duration of analgesia, h	10.3 ± 3.0	10.5 ± 2.7	0.766
Patient satisfaction, 0–10	8 (8–10)	10 (8–10)	0.342
Surgeon satisfaction, 0–10	10 (10–10)	10 (10–10)	0.386
Time to discharge, h	24 (24–30)	24 (21–36)	0.976

Values are median (interquartile range) or mean ± standard deviation according to distribution. Total procedure time was the primary outcome; its effect size is expressed as the Hodges–Lehmann median difference with a 95% confidence interval (CI). CCB, costoclavicular block; ICB, infraclavicular block.

**Table 3:** Postoperative pain intensity (Visual Analog Scale, 0–10).

Time Point	Costoclavicular (*n* = 29)	Infraclavicular (*n* = 31)	*p*
VAS at 6 h	2 (2–3)	2 (2–3)	0.600
VAS at 12 h	4 (3–5)	4 (4–5)	0.635
VAS at 24 h	4 (3–4)	3 (2–4)	0.074

Values are median (interquartile range). Comparisons used the Mann–Whitney U test. VAS, Visual Analog Scale.

**Table 4:** Patients requiring rescue analgesia within each postoperative interval.

Interval	Costoclavicular (*n* = 29)	Infraclavicular (*n* = 31)	*p*
0–6 h	6 (20.7)	5 (16.1)	0.745
6–12 h	19 (65.5)	22 (71.0)	0.783
12–24 h	11 (37.9)	15 (48.4)	0.446

Values are n (%) of patients who received at least one rescue analgesic during the specified interval. Comparisons used Fisher’s exact test. The stepwise rescue protocol is described in Section 2.4.

## Data Availability

The de-identified data supporting the findings of this study are available from the corresponding author upon reasonable request, subject to institutional and ethical restrictions.

## Supplementary Materials

The following supporting information can be downloaded at https://www.mdpi.com/article/10.3390/diagnostics16203274/s1, Table S1: Perioperative hemodynamic and respiratory parameters and operative duration; File S1: STROBE Statement—Checklist of items to be included in reports of cohort studies.

## Abbreviations

ASA	American Society of Anesthesiologists physical status
BMI	body mass index
CCB	costoclavicular block
CI	confidence interval
ICB	infraclavicular block
IQR	interquartile range
MAP	mean arterial pressure
STROBE	Strengthening the Reporting of Observational Studies in Epidemiology
VAS	visual analog scale
