# Supplementary material for: Comparison of Clinical Efficacy and Postoperative Outcomes of Ultrasound-Guided Lateral Sagittal Infraclavicular and Costoclavicular Brachial Plexus Blocks: A Retrospective Cohort Study
Source: Diagnostics (Basel). 2026 Oct 9;16(20):3274. doi: 10.3390/diagnostics16203274 (PMC13703624; doi:10.3390/diagnostics16203274)
Supplement: Supplementary file 1 [file diagnostics-16-03274-s001.zip › Supplementary_File_S1.pdf]

STROBE Statement — Checklist of items to be included in reports of cohort studies

Supplementary File S1 accompanying: "Comparison of Clinical Efficacy and Postoperative Outcomes of Ultrasound-Guided Lateral Sagittal Infraclavicular and Costoclavicular Brachial Plexus Blocks: A Retrospective Cohort Study"

Section and item numbers follow the STROBE (Strengthening the Reporting of Observational Studies in Epidemiology) statement for cohort studies. The final column indicates how each recommendation is addressed in the manuscript.

Item	Section / Topic	STROBE recommendation	How and where addressed in this manuscript
**Title and abstract**			
1	*Title and abstract*	(a) Indicate the study's design with a commonly used term in the title or the abstract.<br>(b) Provide in the abstract an informative and balanced summary of what was done and what was found.	(a) The title identifies the design as a retrospective cohort study. (b) A structured abstract (Background/Objectives, Methods, Results, Conclusions) provides a balanced summary. Title and Abstract.
**Introduction**			
2	*Introduction*	Background/rationale: Explain the scientific background and rationale for the investigation being reported.	The anatomical and ultrasound-guided basis of both approaches and the inconsistent comparative evidence are described. Section 1, paragraphs 1-3.
3	*Introduction*	Objectives: State specific objectives, including any prespecified hypotheses.	The primary objective (total block procedure time) and secondary outcomes are stated. Section 1, final paragraph.
**Methods**			
4	*Methods*	Study design: Present key elements of study design early in the paper.	The retrospective, observational, two-center cohort design is presented at the start of Section 2.1.
5	*Methods*	Setting: Describe the setting, locations and relevant dates, including periods of recruitment, exposure, follow-up and data collection.	The setting (Fırat University Hospital and Bingöl State Hospital), April-October 2025 study period, record sources and 24-h postoperative assessment window are reported. Sections 2.1, 2.2 and 2.4.
6	*Methods*	Participants: (a) Give the eligibility criteria, and the sources and methods of selection of participants; describe methods of follow-up.<br>(b) For matched studies, give matching criteria and number of exposed and unexposed.	Eligibility criteria, record sources and selection methods are described. This was not a matched study; the exposure groups were CCB (n = 29) and ICB (n = 31). Section 2.2 and Figure 1.
7	*Methods*	Variables: Clearly define all outcomes, exposures, predictors, potential confounders and effect modifiers. Give diagnostic criteria, if applicable.	The exposure, primary and secondary outcomes, block success/failure, clinically apparent hemidiaphragmatic paralysis and local anesthetic systemic toxicity, and comparable specialist experience (approximately 4 years) across both centers are described. Sections 2.3-2.4.
8	*Methods*	Data sources/measurement: For each variable of interest, give sources of data and details of methods of assessment (measurement). Describe comparability of assessment methods if there is more than one group.	Structured anesthesia records, cold-sensation testing, the institutional modified motor scale, close continuous specialist observation with exact onset-time recording, safety-record review, and 0-10 VAS/satisfaction scales are described and were applied to both groups. Section 2.4.

Item	Section / Topic	STROBE recommendation	How and where addressed in this manuscript
9	*Methods*	Bias: Describe any efforts to address potential sources of bias.	Selection bias, unblinded clinical measurement, unavailable operator identifiers, center effects, and limited precision for uncommon safety events are identified and carried into the interpretation. Sections 2.2, 2.4, 2.5 and 4.1.
10	*Methods*	Study size: Explain how the study size was arrived at.	No a priori sample-size calculation was performed; all eligible complete records from the fixed retrospective period were analyzed. Section 2.5.
11	*Methods*	Quantitative variables: Explain how quantitative variables were handled in the analyses. If applicable, describe which groupings were chosen and why.	Continuous variables remained continuous and were summarized as mean ± SD or median (IQR) according to distribution. Rescue use was analyzed as a binary per-interval variable. Section 2.5.
12	*Methods*	Statistical methods: (a) Describe all statistical methods, including those used to control for confounding. (b) Describe any methods used to examine subgroups and interactions. (c) Explain how missing data were addressed. (d) If applicable, explain how loss to follow-up was addressed. (e) Describe any sensitivity analyses.	(a) Statistical tests and the Hodges-Lehmann effect estimate are specified; no confounder-adjusted model was fitted. (b) No subgroup/interaction analysis. (c) Complete-case records were analyzed without imputation. (d) No longitudinal loss to follow-up within the 24-h window. (e) No sensitivity analysis. Section 2.5 and Section 4.1.
**Results**			
13	*Results*	Participants: (a) Report numbers of individuals at each stage. (b) Give reasons for non-participation at each stage. (c) Consider use of a flow diagram.	The flow reports 130 screened, 62 eligibility exclusions, 68 blocks performed, 8 clinical exclusions and 60 analyzed patients, with exclusion categories and group distribution of failed blocks/Horner syndrome. Section 2.2, Section 3.1 and Figure 1.
14	*Results*	Descriptive data: (a) Give characteristics of study participants and information on exposures and potential confounders. (b) Indicate number of participants with missing data for each variable. (c) Summarise follow-up time.	Table 1 reports demographics, ASA class, smoking, comorbidity, and surgical site; comparable specialist experience (approximately 4 years) is described in Sections 2.3 and 3.1. The analytic cohort contained complete block-assessment records; no imputation was used. Follow-up covered 24 h.
15	*Results*	Outcome data: Report numbers of outcome events or summary measures over time.	Procedural and block outcomes are reported in Table 2, hemodynamic variables in Supplementary Table S1, VAS scores in Table 3, rescue analgesia in Table 4, and screening-level safety observations in Section 3.4.
16	*Results*	Main results: (a) Give unadjusted estimates and, if applicable, confounder-adjusted estimates and their precision. (b) Report category boundaries when continuous variables were categorized. (c) If relevant, translate relative risk into absolute risk.	Unadjusted group estimates and p values are reported; the primary outcome includes a Hodges-Lehmann median difference with 95% CI. Continuous variables were not categorized, and rescue use is reported as absolute n (%). Section 3 and Tables 1-4.
17	*Results*	Other analyses: Report other analyses done—eg analyses of subgroups and interactions, and sensitivity analyses.	No subgroup, interaction or sensitivity analyses were performed because of the sample size. Section 2.5.
**Discussion**			
18	*Discussion*	Key results: Summarise key results with reference to study objectives.	The opening paragraph of Section 4 summarizes findings in relation to the stated objectives.
19	*Discussion*	Limitations: Discuss limitations of the study, taking into account sources of potential bias or imprecision. Discuss both direction and magnitude of any potential bias.	Section 4.1 addresses non-random allocation, unavailable operator identifiers and center effects, complete-case selection, modest study size, uncommon safety events, wide age range, unblinded clinical assessment, and applicability only to the standardized fixed-dose 20-mL regimen.

Item	Section / Topic	STROBE recommendation	How and where addressed in this manuscript
20	*Discussion*	Interpretation: Give a cautious overall interpretation of results considering objectives, limitations, multiplicity of analyses, results from similar studies, and other relevant evidence.	Interpretation is cautious, considers prior trials and meta-analysis, separates the unadjusted procedural signal from the analgesic findings and avoids equivalence claims. Sections 4 and 5.
21	*Discussion*	Generalisability: Discuss the generalisability (external validity) of the study results.	External validity is discussed in relation to the two-center routine-practice setting, comparable specialist experience with both techniques (approximately 4 years), the wide age range, the standardized fixed-dose regimen, and the complete-case cohort. Section 4.1.
**Other information**			
22	*Other information*	Funding: Give the source of funding and the role of the funders for the present study and, if applicable, for the original study on which the present article is based.	The study received no external funding. Funding statement.

The STROBE checklist is available from the STROBE Initiative (www.strobe-statement.org). This completed checklist should be read together with the main manuscript.
