# Supplementary material for: Comparison of Clinical Efficacy and Postoperative Outcomes of Ultrasound-Guided Lateral Sagittal Infraclavicular and Costoclavicular Brachial Plexus Blocks: A Retrospective Cohort Study
Source: Diagnostics (Basel). 2026 Oct 9;16(20):3274. doi: 10.3390/diagnostics16203274 (PMC13703624; doi:10.3390/diagnostics16203274)
Supplement: Supplementary file 1 [file diagnostics-16-03274-s001.zip › Supplementary_Table_S1.pdf]

Supplementary Material

Table S1. Perioperative hemodynamic and respiratory parameters and operative duration

Variable	Costoclavicular (n = 29)	Infraclavicular (n = 31)	p
Heart rate – preoperative, bpm	78.6 ± 14.7	77.1 ± 9.7	0.970
Heart rate – intraoperative, bpm	77.8 ± 13.6	78.3 ± 13.9	0.970
Heart rate – postoperative, bpm	76.1 ± 12.8	76.9 ± 13.5	0.853
Mean arterial pressure – preoperative, mmHg	95.7 ± 15.1	97.9 ± 12.8	0.407
Mean arterial pressure – intraoperative, mmHg	91.2 ± 12.4	99.1 ± 12.2	0.012
Mean arterial pressure – postoperative, mmHg	88.9 ± 11.9	99.0 ± 13.5	0.003
Peripheral oxygen saturation – preoperative, %	96.5 ± 2.8	96.1 ± 2.6	0.416
Peripheral oxygen saturation – intraoperative, %	97.6 ± 2.4	97.0 ± 2.5	0.189
Peripheral oxygen saturation – postoperative, %	97.1 ± 2.8	96.8 ± 2.6	0.549
Operative duration, min	40 (30–70)	40 (25–77)	0.965

Values are mean ± standard deviation, except operative duration, which is shown as median (interquartile range). Comparisons used the independent-samples t test or Mann-Whitney U test, as appropriate. bpm, beats per minute; CCB, costoclavicular block; ICB, infraclavicular block.
